# Differential upregulation in DRG neurons of an α_2_δ-1 splice variant with a lower affinity for gabapentin after peripheral sensory nerve injury

**DOI:** 10.1016/j.pain.2013.12.001

**Published:** 2014-03

**Authors:** Beatrice Lana, Bettina Schlick, Stuart Martin, Wendy S. Pratt, Karen M. Page, Leonor Goncalves, Wahida Rahman, Anthony H. Dickenson, Claudia S. Bauer, Annette C. Dolphin

**Affiliations:** Department of Neuroscience, Physiology and Pharmacology, University College London, London, UK

**Keywords:** Gabapentin, Neuropathic pain, Calcium channel, Splice variant, Alpha2delta (α_2_δ)

## Abstract

The α_2_δ-1 protein is an auxiliary subunit of voltage-gated calcium channels, critical for neurotransmitter release. It is upregulated in dorsal root ganglion (DRG) neurons following sensory nerve injury, and is also the therapeutic target of the gabapentinoid drugs, which are efficacious in both experimental and human neuropathic pain conditions. α_2_δ-1 has 3 spliced regions: A, B, and C. A and C are cassette exons, whereas B is introduced via an alternative 3′ splice acceptor site. Here we have examined the presence of α_2_δ-1 splice variants in DRG neurons, and have found that although the main α_2_δ-1 splice variant in DRG is the same as that in brain (α_2_δ-1 ΔA+B+C), there is also another α_2_δ-1 splice variant (ΔA+BΔC), which is expressed in DRG neurons and is differentially upregulated compared to the main DRG splice variant α_2_δ-1 ΔA+B+C following spinal nerve ligation. Furthermore, this differential upregulation occurs preferentially in a small nonmyelinated DRG neuron fraction, obtained by density gradient separation. The α_2_δ-1 ΔA+BΔC splice variant supports Ca_V_2 calcium currents with unaltered properties compared to α_2_δ-1 ΔA+B+C, but shows a significantly reduced affinity for gabapentin. This variant could therefore play a role in determining the efficacy of gabapentin in neuropathic pain.

## Summary

A minor α_2_δ-1 splice-variant, (ΔA+BΔC), with reduced affinity for gabapentin, is differentially upregulated in DRG neurons following ligation. This could influence the efficacy of gabapentin.

## 1 Introduction

Voltage-gated Ca^2+^ channels of the Ca_V_1 and Ca_V_2 families contain 3 subunits: the pore-forming α1 subunit, together with 2 auxiliary subunits, β and α_2_δ, both of which increase the functional expression of the channels [4], [11], [13], [20], [21]. The α_2_δ subunits are each the product of a single gene (*CACNA2D1-4*), encoding an α_2_δ preprotein, which is posttranslationally processed into α_2_ and δ [22]. We have recently shown that α_2_δ subunits can form glycosylphosphatidylinositol-anchored proteins [19], which are constitutively endocytosed and reinserted into the plasma membrane via the recycling endosomes [5], [48]. We have identified that the mechanism whereby α_2_δ-1 and -2 subunits enhance plasma-membrane expression of calcium channels involves the metal ion-dependent adhesion site (MIDAS) motif in their von Willebrand factor A (VWA) domain [12], [31], and it is also important for the enhancement of presynaptic vesicular release [31].

The α_2_δ-1 subunit has a widespread distribution; both mRNA and protein are found in neuronal tissue, heart, skeletal, and smooth muscle [3]. In dorsal root ganglion (DRG) neurons, α_2_δ-1 is the main α_2_δ subunit expressed [5], [16], [36]. The α_2_δ-1 protein is upregulated following various types of peripheral nerve injury [5], [39], [49], and this upregulation is essential for the rapid development of the subsequent behavioural mechanical hypersensitivity seen in animal models [41]. By contrast, Ca_V_2.2 mRNA and protein are not reported to be consistently upregulated following sensory nerve damage [1], [36], [49], suggesting that upregulated α_2_δ-1 protein enhances Ca_V_2.2 trafficking and presynaptic function.

The α_2_δ-1 and α_2_δ-2 subunits bind to the gabapentinoid drugs, gabapentin and pregabalin. These were developed as antiepileptic drugs and are also widely used in the treatment of various forms of neuropathic pain [46]. The α_2_δ-1 subunit has been shown to represent the target for these drugs in the alleviation of hyperalgesia in experimental models of neuropathic pain [24]. We have found that gabapentin and pregabalin reduce the trafficking of α_2_δ subunits both *in vitro* and *in vivo* [5], [30], [48], which is likely to represent one of their main mechanisms of action. Furthermore, α_2_δ-1 has been reported to interact with thrombospondins, a family of extracellular matrix proteins, and this may influence its trafficking as well as the effect of gabapentinoid drugs [23].

**Fig. 1:**
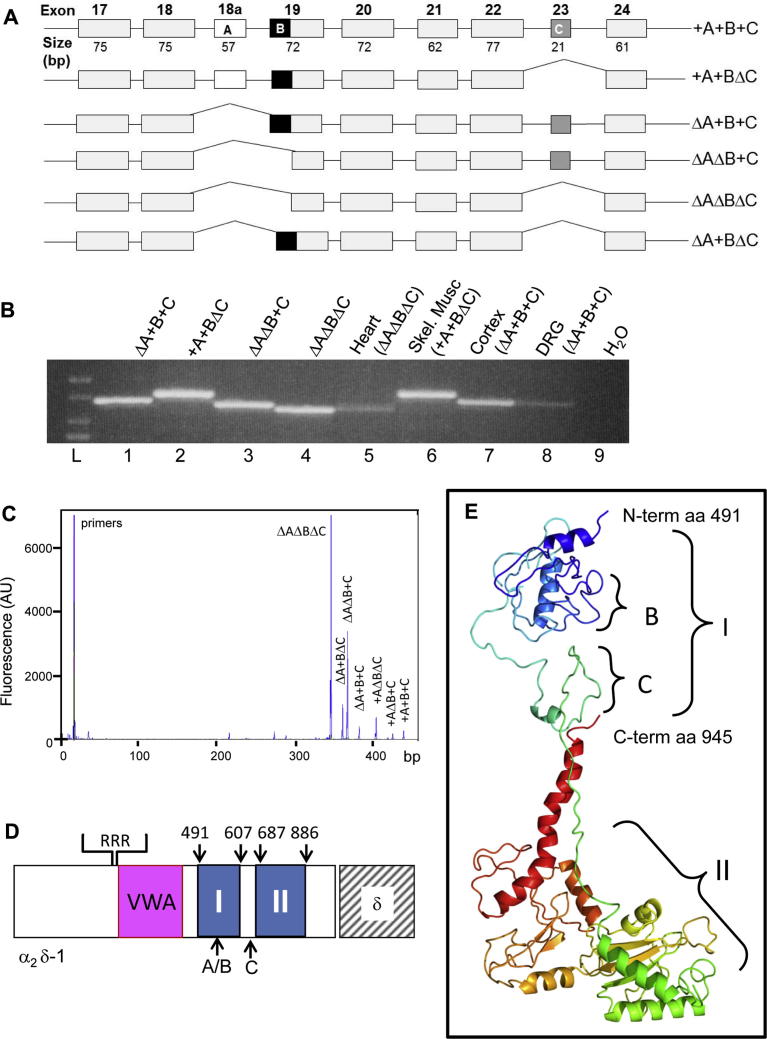
Distribution of different α_2_δ-1 splice variants in rat tissue. (A) Diagram of possible splice variants of α_2_δ-1, showing arrangement of exons within the region of alternative splicing, and position of cassette exon 18a encoding A (white), alternative spliced region B (black), and cassette exon 23 encoding C (dark grey), data obtained from Ensembl database, gene IDs ENSG00000153956 (human), ENSMUSG00000040118 (mouse), and ENSRNOG00000033531 (rat). (B) Identification of α_2_δ-1 splice variants using outer primer pairs. Bands were recovered from the gel and sequenced, to verify identification. Lanes 1–4 were amplified from plasmids, lanes 5–8 were amplified from the tissues stated, lane 9 is the water control, and lane L is the size ladder. Samples were run on 2% MetaPhor Agarose Gel (Cambrex Bio Science Rockland, Inc., Rockland, ME, USA) in Tris Acetate Ethylenediaminetetraacetic acid, 100V. (C) ΔAΔBΔC is the most abundant α_2_δ-1 splice variant in rat heart. Representative electropherogram of capillary electrophoresis/laser-induced fluorescence with polymerase chain reaction (PCR) products derived from rat heart. Reverse-transcription PCR was performed for 30 cycles on 100 ng of total RNA equivalent. α_2_δ-1 transcript products were separated by size along the *x*-axis: ΔAΔBΔC (345 bp), ΔA+BΔC (360 bp), ΔAΔB+C (366 bp), ΔA+B+C (381 bp), +AΔBΔC (402 bp), +AΔB+C (423 bp), +A+B+C (438 bp). The *y*-axis indicates the fluorescence signal peak height, which corresponds to the expression level of the respective splice variant. (D) Diagram of domains in α_2_δ-1 to show the approximate positions of the 2 domains with homology to bacterial chemosensory domains (CSDs), with respect to the von Willebrand factor A domain and to regions A, B, and C. (E) Structure prediction for the bacterial chemosensor-like domains of α_2_δ-1. The sequence of α_2_δ-1 including the 2 predicted bacterial chemosensory domains (starting at amino acid 491[Asp-Val-Ser-Leu …]-945 (… Leu-Glu-Ala, which is the end of α_2_-1) was submitted to Phyre2 [33] for structure prediction (http://www.sbg.bio.ic.ac.uk/phyre2). The amino acid numbering includes the N-terminal signal sequence of α_2_δ-1. Region I (∼amino acids 491–607) was predicted with ∼99% confidence, and modelled on a number of bacterial CSDs, including the extracellular domain of the *Bacillus subtilis* CSD (mmhk1s-z2) and the putative sensory box/ggdef protein from *Vibrio parahaemolyticus*. Region II (∼amino acids 687–886) was modelled with a predicted ∼98% confidence on a separate subset of CSDs, including the C4-dicarboxylate transport sensor protein dctb, and the mcp_n and cache domains of methyl-2 accepting chemotaxis protein from *Vibrio cholerae*. The regions between domains I and II and beyond II are not modelled with high confidence. The approximate positions of the two spliced regions B and C are indicated. They are both likely to be in exposed loops, B being within the first chemosensory domain, and C being between the 2 domains. A is not present in this model.

Alternatively spliced isoforms of α_2_δ-1 in different tissues have been observed previously [10], [22]. It was reported that the mouse *cacna2d1* gene has 3 alternatively spliced regions (A, B, and C), and 5 splice variants were identified [3] (Fig. 1A). In the present study we have examined the hypothesis that there may be differential upregulation of specific splice variants of α_2_δ-1 following peripheral nerve damage, and that this might potentially contribute to either the state dependency [14], [25] or to limiting the response to gabapentinoids, as these drugs are found to have variable efficacy in patients, with number-needed-to-treat values of about 4–5 [37], [38].

## 2 Methods

### 2.1 Molecular biology and heterologous expression of cDNAs

Rat α_2_δ-1 (M86621.3) [47], which encodes the main brain splice variant ΔA+B+C, was used as the starting point for assembly of the α_2_δ-1 splice variants used in this study. All mutations were made by standard molecular biological techniques, and verified by DNA sequencing. Other calcium channel complementary DNAs (cDNAs) used were rat Ca_V_2.1 (M64373), rabbit Ca_V_2.2 (D14157), and rat β1b [47]. The cDNAs were cloned into the pMT2 vector for expression. For heterologous expression, tsA-201 cells were transfected with the cDNA combinations stated. The cDNA for green fluorescent protein (mut3 GFP) [17] was also included to identify transfected cells from which electrophysiological recordings were made. Transfection was performed as described previously [40]. In control experiments where α_2_δ was omitted, the ratio was made up with empty vector.

### 2.2 Isolation of detergent-resistant membranes (DRMs)

This procedure was performed as previously described [19], [32]. Briefly, confluent cells from 6 175-cm^2^ flasks (72 hours after transfection), or pelleted homogenate from rat whole brain, were resuspended in 1.5 mL 2-(*N*-morpholino)ethanesulfonic acid (MES)-buffered saline containing 1% (2% for brain tissue) Triton X-100 (Perbio, Tattenhall, Cheshire, UK) and left on ice for 1 hour. After the addition of an equal volume of 90% (w/v) sucrose, the sample was overlaid with a 10-mL discontinuous sucrose gradient and centrifuged at 140,000 × g for 18 hours at 4°C. One-mL fractions of the gradient were harvested from the top to the bottom of the tube, washed free of sucrose by dilution in MES-buffered saline and subsequent centrifugation. The detergent-resistant membranes (DRMs) were prepared from pooled fractions 4, 5, and 6. The resulting pellet was resuspended in 4-(2-hydroxyethyl)-1-piperazineethanesulfonic acid (HEPES) 10 mM pH 7.4 and complete protease inhibitor cocktail (Roche Diagnostics GmbH, Mannheim, Germany). The isolated DRM fractions were collected for Western blot analysis and used in the ^3^H-gabapentin binding assay.

### 2.3 Immunoblotting

Western blotting was performed as described previously [40]. The following primary antibodies were used: anti-α_2_-1 (1:1000; mouse monoclonal, Sigma-Aldrich, St. Louis, MO, USA) and anti-flotillin-1 (1:2000; mouse monoclonal, BD Biosciences, Franklin Lakes, NJ, USA). The secondary antibody used was goat anti-mouse coupled to horseradish peroxidase (Bio-Rad Laboratories, Inc., Hercules, CA, USA). Protein bands were quantified using Image J software (rsb.info.nih.gov). All α_2_δ-1 splice variant expression levels were corrected for background and normalized to ΔA+B+C protein expression on the same gel.

### 2.4 ^3^H gabapentin binding assay

Binding of ^3^H-gabapentin to DRM fractions from transfected tsA-201 cells was carried out in a final volume of 250 μL at room temperature for 45 minutes. DRMs (4 μg protein per tube) were incubated with various concentrations of ^3^H-gabapentin [45] (30–50 Ci/mmol, American Radiolabeled Chemicals, St. Louis, MO, USA) in 10 mM HEPES/KOH pH 7.4, then rapidly filtered through glass fibre (B grade, Whatman) filters presoaked with 0.3% polyethyleneimine. Filters were washed 3 times with 3 mL ice-cold 100 mM Tris/HCl pH 7.4 and counted in a scintillation counter. Concentrations of ^3^H-gabapentin >10 nM were achieved by adding nonradioactive gabapentin and correcting the specific binding by the dilution factor [12]. Nonspecific binding was determined in the presence of 100 μM nonradioactive gabapentin. Data points were determined in triplicate, and data were analysed by fitting specific binding to the equation for a rectangular hyperbola.

### 2.5 Spinal nerve ligation (SNL)

A total of 17 male Sprague-Dawley rats (Central Biological Services, University College London, London, UK) weighing 130–150 g at time of surgery were employed for this study. All experimental procedures were approved by the UK Home Office and followed the guidelines of the International Association for the Study of Pain [51]. Selective spinal nerve ligation (SNL) surgery was conducted as previously described [34]. Briefly, the left L5 and L6 spinal nerves were isolated and tightly ligated with 6-0 silk thread under isoflurane anaesthesia (50% O_2_: 50% N_2_O). Haemostasis was confirmed and the wound was sutured. After surgery the animals were allowed to recover and housed at a maximum of 5 per cage. Food and water were available ad libitum. The foot posture and general behaviour of the operated rats were monitored throughout the postoperative period, and the development of mechanical hypersensitivity was confirmed at 7 days post surgery in the affected limb ipsilateral to the ligation, as previously described [5].

### 2.6 Enrichment of small and large DRG neurons using density gradient centrifugation

In order to obtain sufficient material, both L5 and L6 DRG were extracted from the ipsilateral and contralateral sides of 2 rats, either naïve animals or 7 days after SNL. Nerve roots were trimmed and the isolated ganglia were incubated in 5 U/mL of collagenase (Sigma-Aldrich) in 2 mL of Hanks balanced saline solution in a shaking water bath at 37°C for 30 minutes, and then triturated. The cell suspension was sedimented at 4°C for 5 minutes at 200 × g. The pellet was resuspended in culture medium composed of Dulbecco’s Modified Eagle’s Medium: Nutrient Mixture F-12 (Gibco, Invitrogen, Life Technologies, Grand Island, NY, USA), penicillin (100 U/mL), streptomycin (100 μg/mL), l-glutamine, (2 mM), nerve growth factor (100 ng/mL, Sigma-Aldrich), and 10% foetal calf serum. Four mL of DRG neurons in culture medium was filtered through a 100-μm cell strainer (BD Biosciences) and layered onto 5 mL of a solution of 40% Ficoll (Histopaque-1077, Sigma-Aldrich) and 60% phosphate-buffered saline containing 4.2 mM NaHCO_3_. The final pH was adjusted to 7.3–7.4 with HCl. The preparation was centrifuged at 100 × g for 15 minutes at 4°C, and DRG neurons were separated according to their size into a low-density fraction (LDF) and a high-density fraction (HDF) [28]. The 2 fractions obtained were diluted to 10 mL with culture medium and then centrifuged at 200 × g for 5 minutes at 4°C. The LDF and HDF pellets, enriched in viable small and large neurons, respectively, were obtained and immediately used for RNA extraction, or plated on 13-mm-diameter poly-d-lysine-coated (10 mg/mL; Sigma-Aldrich) glass coverslips. Cells were incubated at 37°C (95% air, 5% CO_2_), and observed in bright field after 2–5 hours to measure the density and diameter of the neurons in each fraction. Only phase-bright cells with a typical DRG morphology (large, clear nucleus with prominent nucleolus) were considered for statistical analysis. Larger cells were often slightly elliptical in cross-section, and the diameter was taken as the mean of the major and minor axes. Quantification of neurofilament-200 (NF-200) mRNA, a well-established marker for medium to large neurons [26], [29] was employed to confirm the successful separation of neuronal fractions.

### 2.7 Quantification of splice variants and NF-200 by quantitative polymerase chain reaction (PCR) and capillary electrophoresis (CE)

RNA isolation and reverse-transcription polymerase chain reaction (RT-PCR) was carried out as follows. Total RNA was extracted from individual L4 or L5 pulverized frozen DRG 7 days after SNL or sham surgery, and pelleted LDF and HDF after density gradient centrifugation. RNA was isolated using the RNeasy Protect Mini Kit (Qiagen, GmbH, Hilden, Germany). RNA concentrations and purity were determined spectrophotometrically. RT was performed on 1 μg of total RNA using Superscript III reverse transcriptase (Invitrogen, Carlsbad CA, USA), using random hexamer primers (Promega, Madison WI, USA) and RNaseOUT (Invitrogen).

In order to quantify the α_2_δ-1 splice variant expression pattern in rat tissue (Fig. 1) and in DRG neurons following SNL, gene-specific primers for rat α_2_δ-1 (Eurofins MWG Operon, Ebersberg, Germany) were designed flanking all 3 splicing sites. For the analysis of the α_2_δ-1 splice variant expression pattern in LDF and HDF, gene-specific primers for NF-200 and α_2_δ-1, flanking only region C, were designed. TATA-box binding protein (TBP) was co-amplified in each reaction and served as an internal control in all experiments. The primer combinations resulted in the following target-specific PCR products: 145 bp (TBP), 360 bp (α_2_δ-1; splice variant ΔA+BΔC), 381 bp (α_2_δ-1; splice variant ΔA+B+C), 101 bp (NF-200), 154 bp (α_2_δ-1; splice variant lacking region C), and 175 bp (α_2_δ-1; splice variant with region C). Either forward or reverse primers were labelled with 6-carboxyfluorescein. PCR was performed on 100 ng (whole DRG), 8 ng (for α_2_δ-1 in LDF and HDF), and 3 ng (for NF-200 in LDF and HDF) of total RNA equivalent, with Taq DNA Polymerase (Bioline Ltd., London, UK) in a total of 20 μL reaction volume. Thermal cycling was conducted with the following conditions: denaturation at 95°C for 2 minutes; 27, 28, or 29 cycles of 95°C for 30 seconds, 65°C for 30 seconds, and 72°C for 40 seconds. The following PCR primers were used: rat α_2_δ-1 (NM_012919), 5′-CGATCCTAATGGTTATGTCTTACTGC-3′ (forward) and 5′-TCAAAATTGTCTGGCTTCAGGGTTTCT-3′ (reverse) flanking all 3 splicing sites; α_2_δ-1_C 5′-GTCAATGGCACAGATTACAG-3′ (forward), α_2_δ-1_C 5′-GTGCTATGAAAGTGTAGCCAG-3′ (reverse) flanking only region C; rat NF-200 (NM_012607) 5′-AAAGTGAACACGGATGCTATG C-3′ (forward), NF-200 5′-GTGCTTTTCAGTGCCTCCAAC-3′ (reverse); rat TBP (NM_001004198), 5′-GGATTGTACCACAGCTCCAAAATAT-3′ (forward), 5′-CGTGGCTCTCTTATTCTCATGATG-3′ (reverse). Primers were designed either manually or using the open-source software Primer3. The techniques were optimized in a series of preliminary experiments for cycle number and starting quantity to ensure points of measurement were acquired during the linear phase of amplification (Supplementary Fig. 1, and data not shown).

Capillary electrophoresis (CE) was carried out following dilution (1:20 for α_2_δ-1 primers flanking all splice regions; 1:5 for NF-200) with deionized water of aliquots of RT-PCR products, and further analysed by laser-induced fluorescence (LIF). A detailed description and validation of the CE/LIF technique used for quantitative analysis of RT-PCR products was reported previously [43]. One μL of sample was diluted with 12 μL HiDi-Formamide (Applied Biosystems, Foster City CA, USA), and 0.5 μL of GeneScan 400HD ROX Size Standard (Applied Biosystems). Amplified PCR products were separated on an ABI 3100 *Avant* Genetic Analyzer (Applied Biosystems) running a 50-cm capillary with 3100 POP-6 polymer (Applied Biosystems). Each sample was injected in triplicate from separate wells on the plate. The amplified products were sized and quantified in GeneMapper v3.5 software using the Local Southern method. This analysis method was chosen for its reciprocal relationship between fragment size and mobility.

The relative abundance of mRNA was determined as the ratio of integrated peak area for each PCR product relative to that of co-amplified TBP in order to allow a direct comparison between different preparations. Then, data from the ipsilateral side were normalized to their respective contralateral side. The percentage of the total transcript represented by the minor DRG isoform (ΔA+BΔC) was calculated as 100∗ pa_ΔA+BΔC_/(pa_ΔA+B+C_+pa_ΔA+BΔC_) [44]. Data were analysed using Microsoft Excel (Microsoft Corporation, Redmond, WA, USA) and SPSS statistical software (SPSS Inc, Chicago IL, USA) or GraphPad Prism 4 (GraphPad Software, San Diego, CA, USA). Statistical significance was determined using the nonparametric Mann-Whitney *U* test and one-way analysis of variance with a suitable post hoc analysis. *P* values <0.05 were considered as statistically significant. All data are presented as mean ± SEM for the indicated number of experiments.

### 2.8 Electrophysiology

Calcium channel expression in tsA-201 cells was investigated by whole-cell patch clamp recording, essentially as described previously [7], using an Axopatch 1D amplifier (Axon Instruments, Burlingame, CA, USA). Recordings were made 2 days after transfection. Pipettes of resistance 2–4 MΩ were used, and the internal (pipette) and external solutions and recording techniques were similar to those previously described [30]. The patch pipette solution contained (in mM): Cs-aspartate, 140; ethylene glycol tetraacetic acid, 5; MgCl_2_, 2; CaCl_2_, 0.1; K_2_ATP, 2; HEPES, 20; pH 7.2, 310 mOsm with sucrose. The external solution for recording Ba^2+^ currents contained (in mM): tetraethylammonium Br, 160; KCl, 3; NaHCO_3_, 1.0; MgCl_2_, 1.0; HEPES, 10; glucose, 4; BaCl_2_, 1, pH 7.4, 320 mOsm with sucrose. Data were filtered at 1–2 kHz and digitized at 5–10 kHz. Current records were subjected to leak and residual capacitance current subtraction (P/8 protocol). Analysis was performed using Pclamp 9 (Molecular Devices, Sunnyvale, CA, USA) and Origin 7 (Microcal Origin, Northampton, MA, USA).

## 3 Results

### 3.1 Distribution of different splice variants of α_2_δ-1 in rat tissue

Splice variants of α_2_δ-1 containing the 3 alternatively spliced regions (A, B, and C) were identified previously in mouse tissue [3]. In that study it was reported that regions A and B were part of the same exon, with alternative 3′ splice acceptor sites, which spliced region A in or out [3]. However, the currently available human, mouse, and rat genomic sequences from Ensembl, ENSG00000153956 (human), ENSMUSG00000040118 (mouse), and ENSRNOG00000033531 (rat), indicate that regions A and B are in separate exons, with region A in rat being encoded by exon 18a, and B representing an alternative 3′ splice acceptor site (start site) of exon 19. A diagram of potential splice variants is given in Fig. 1A.

The following cDNAs were assembled by standard molecular biological techniques in the rat α_2_δ-1 backbone: +A+B+C, +A+BΔC, ΔA+B+C, ΔAΔB+C, ΔAΔBΔC, and ΔA+BΔC. All constructs gave rise to products of the expected size with the PCR primers used in this study (Fig. 1B and data not shown). We then examined their distribution in adult rat tissues. ΔAΔBΔC was found to be the main splice variant in rat heart, but a number of other splice variants were also identified by the much more sensitive CE/LIF, demonstrating that this method can be used for identifying and quantifying α_2_δ-1 splice variants (Fig. 1B, C). We found +A+BΔC in skeletal muscle and ΔA+B+C in cerebral cortex (Fig. 1B), as shown previously for mouse tissues [3]. We also found the same brain splice variant, ΔA+B+C, in DRG (Fig. 1B).

### 3.2 Location of the spliced regions within α_2_δ-1

The α_2_δ subunits contain 2 domains with homology to bacterial extracellular chemosensory domains (CSDs) or Cache domains [2], which are downstream of the VWA domain. Bacterial extracellular CSDs bind a variety of nutrients and are generally associated with an intracellular histidine kinase signalling complex [15]. The plant ethylene receptor ETR1 is also a member of this family [15]. In α_2_δ-1, the first of these CSDs is situated between amino acids ∼491–607 [21]. Fig. 1D gives the approximate locations of the spliced regions, and Fig. 1E [33] illustrates a homology model of the 2 CSDs, showing that regions B and C are situated within a loop in the first CSD of rat α_2_δ-1 (I), and in the linker between CSD I and II, respectively. The model in Fig. 1E was generated using a sequence from which region A was absent, but A would be situated just upstream of region B. It is possible that within α_2_δ subunits, these domains might be important for their ability to bind small ligands, including gabapentin [21].

### 3.3 Determination of ^3^H-gabapentin binding affinity for α_2_δ-1 splice variants

**Fig. 2:**
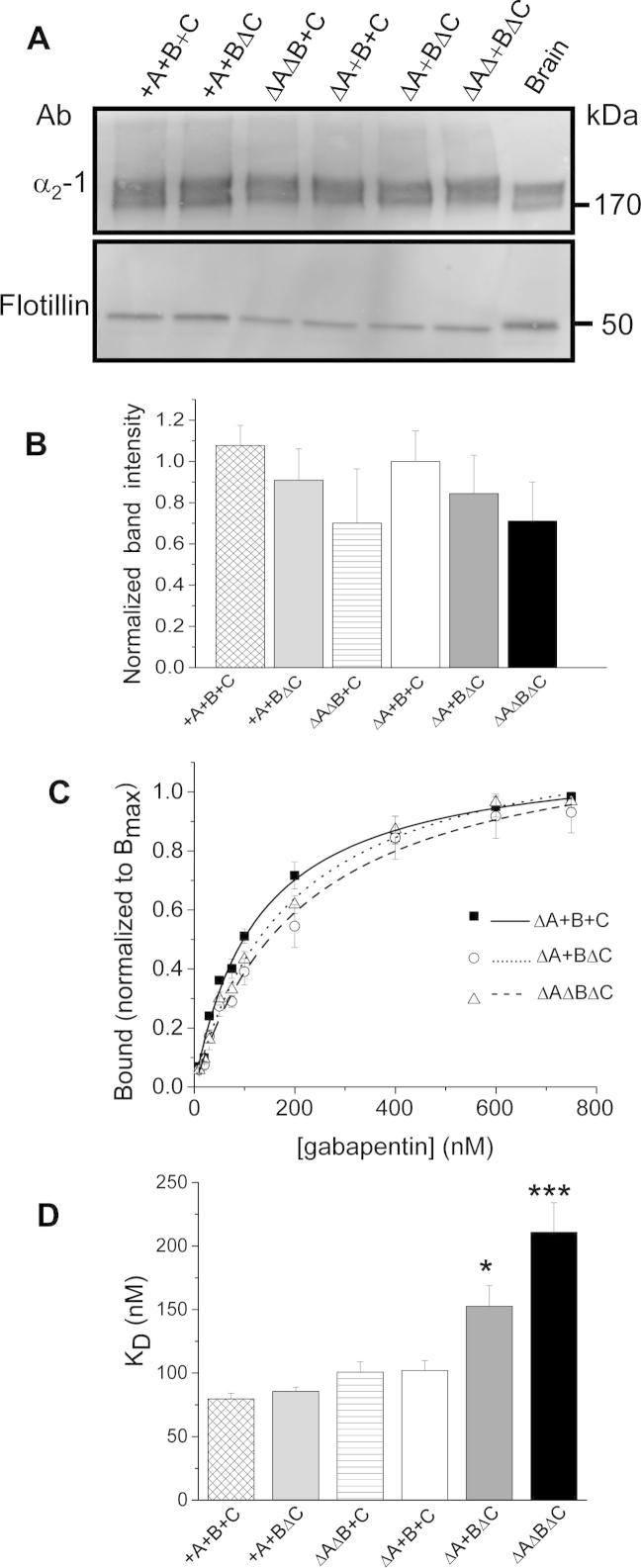
Analysis of the level of expression and gabapentin binding properties of the α_2_δ-1 splice variants. (A) Representative Western blots showing that the level of protein expression in detergent-resistant membrane (DRM) preparations is similar between all the α_2_δ-1 splice variants analysed. (B) No significant difference in mean normalized immunoreactivity of the α_2_δ-1 bands was observed after image analysis (+A+B+C, n = 3; +A+BΔC, n = 3; ΔAΔB+C, n = 3, ΔA+B+C, n = 10; ΔA+BΔC, n = 4, and ΔAΔBΔC, n = 3). Bands were normalized to the main brain splice variant ΔA+B+C in every experiment. Error bars represent SEM. Statistical analysis was performed using one-way analysis of variance (ANOVA) and Bonferroni post hoc analysis. (C) Measurement of the affinity of ^3^H-gabapentin binding sites in DRM fractions for 3 α_2_δ-1 splice variants. The curves show the mean data obtained for α_2_δ-1 ΔA+B+C (■, fitted with solid line), ΔA+BΔC (Δ, fitted with dotted line) and ΔAΔBΔC (○, fitted with dashed line) binding to ^3^H-gabapentin, fitted using the Hill-Langmuir equation. Data were normalized to each mean B_max_ to illustrate the difference in K_D_ values. (D) The mean K_D_ for all the splice variants tested (+A+B+C, n = 5; +A+BΔC, n = 5; ΔAΔB+C, n = 5; ΔA+B+C, n = 12; ΔA+BΔC, n = 8, and ΔAΔBΔC, n = 5). Mean values for K_D_ were calculated from fitting individual experiments, each carried out in triplicate. The bar chart shows the reduced affinity of α_2_δ-1 ΔA+BΔC (dark grey bar) and ΔAΔBΔC (black bar) compared to the predominant brain and dorsal root ganglion splice variant ΔA+B+C (white bar). Error bars represent SEM. Statistical analyses were performed using one-way ANOVA, with Bonferroni post hoc analysis: ^∗^*P* < 0.05, ^∗∗∗^*P* < 0.001. The B_max_ values were not altered, being (in pmol/mg protein) 15.6 ± 2.6 for +A+B+C; 12.1 ± 0.5 for +A+BΔC; 11.8 ± 1.6 for ΔAΔB+C; 15.7 ± 2.9 for ΔA+B+C; 17.0 ± 4.8 for ΔA+BΔC; and 14.7 ± 2.7 for ΔAΔBΔC.

We then examined whether there were differences in ^3^H-gabapentin binding affinity between the different α_2_δ-1 splice variants (Fig. 2). All determinations were performed on concentrated DRM fractions following expression in tsA-201 cells, since we have shown previously that there is a large increase in apparent affinity of both α_2_δ-1 and α_2_δ-2 for ^3^H-gabapentin in DRMs [18], [30].

To ensure that the radioligand binding experiments were not influenced by differential expression of α_2_δ-1 protein between the different splice variants used, the relative expression level in DRMs of each splice variant was analysed by Western blot. All splice variants partitioned similarly into DRM fractions, and their level of expression was also found to be similar (Fig. 2A, B). We found that both ΔAΔBΔC (one of the splice variants found in heart) and ΔA+BΔC showed significantly lower affinity for ^3^H-gabapentin (K_D_ values of 211 and 153 nM, respectively), compared to the major brain and DRG splice variant ΔA+B+C (Fig. 2C, D). Both these splice variants with lower affinity lacked region C, whereas all the splice variants containing region C, including ΔA+B+C, showed a higher affinity for ^3^H-gabapentin, with K_D_ values between 80 and 102 nM (Fig. 2C, D). Thus, there was a nearly 3-fold difference in affinity between the splice variants with the highest and lowest ^3^H-gabapentin binding affinities. In contrast, all splice variants showed similar B_max_ values, indicating that the number of binding sites was not affected (see legend to Fig. 2).

### 3.4 Do novel splice variants of α_2_δ-1 become expressed in DRG following SNL?

Gabapentinoid drugs are used to treat neuropathic pain, so we wondered whether there were changes in splice variant expression in DRG following SNL, which could potentially influence the efficacy of these drugs. We chose 7 days after SNL as the time point for our analyses because we found previously that the increase in α_2_δ-1 mRNA was not significantly different between 7 and 14 days after SNL, and we also noted less variability in the increase in α_2_δ-1 mRNA at 7 than at 14 days [5].

**Fig. 3:**
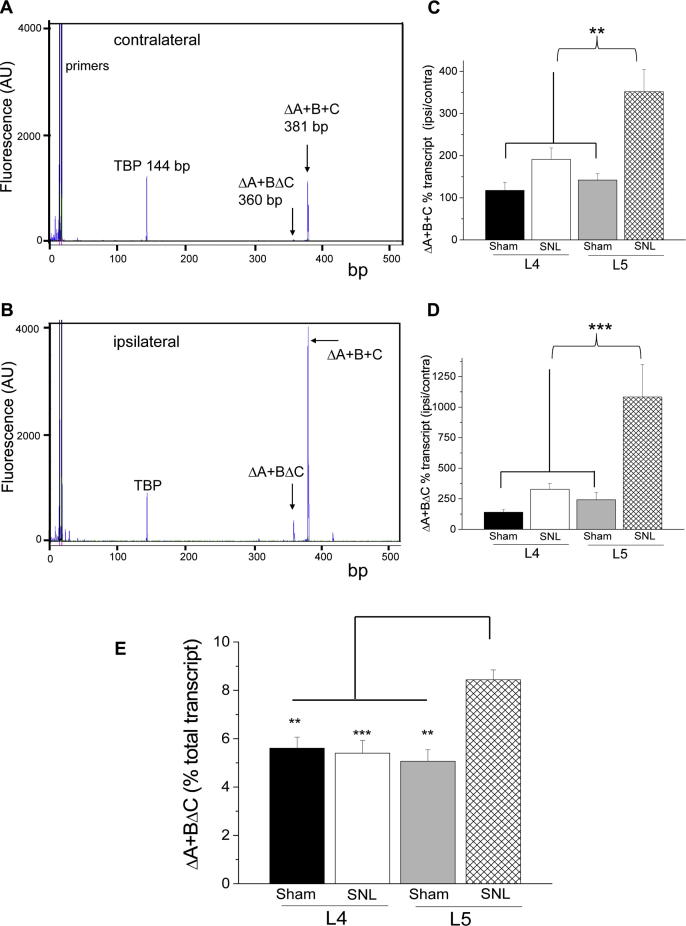
The effect of spinal nerve ligation (SNL) on mRNA levels of the α_2_δ-1 splice variants. (A, B) Representative electropherograms of capillary electrophoresis/laser-induced fluorescence with polymerase chain reaction products derived from L5 dorsal root ganglia (DRG) 7 days after SNL. Transcript products were separated by size along the *x*-axis. The *y*-axis indicates the fluorescence signal peak height, which corresponds to the expression level of the respective splice variant. mRNA levels of ΔA+B+C and ΔA+BΔC are higher in the side ipsilateral to SNL (B) than in the contralateral side (A). Note that the expression of the housekeeping gene (TATA-box binding protein) remains at a constant level on both sides. (C) SNL (7 days) leads to increased expression of the major α_2_δ-1 (ΔA+B+C) splice variant in L5 DRG. Data are expressed as percentage of relative peak areas of the ipsilateral side normalized to the respective contralateral side. The relative increase is shown in L4 DRG from sham-operated rats (black bar, n = 6), L4 DRG from L5/L6 SNL rats (white bar, n = 11), L5 DRG from sham-operated rats (grey bar, n = 6), and L5 DRG after SNL (cross-hatched bar, n = 7). There was no significant difference in L4 DRG between SNL and sham-operated animals. Error bars represent SEM. Analysis of variance (ANOVA), F = 9.012, *P* < 0.0001, and Gabriel post hoc analysis: ^∗∗^*P* < 0.01. (D) A pronounced upregulation of the α_2_δ-1 splice variant ΔA+BΔC was observed in ipsilateral L5 DRG 7 days after SNL. Data are expressed as percentage of relative peak areas of the ipsilateral side normalized to the respective contralateral side. The relative increase is shown in L4 DRG from sham-operated rats (black bar, n = 6), L4 DRG from SNL rats (white bar, n = 11), L5 DRG from sham-operated animals (grey bar, n = 6) and L5 DRG after SNL (cross-hatched bar, n = 7). Although ΔA+BΔC mRNA levels are 2.4-fold higher in SNL L4 compared to L4 sham-operated animals, this was not statistically significant (*P* = 0.814). Error bars represent SEM. ANOVA, F = 11.12, *P* < 0.0001, and Gabriel post hoc analysis: ^∗∗∗^*P* = 0.001. (E) Comparison of the percentage of total transcript represented by ΔA+BΔC splice variant in DRG ipsilateral to SNL, in L4 from sham-operated rats (black bar, n = 6), L4 DRG from SNL rats (white bar, n = 11), L5 DRG from sham-operated animals (grey bar, n = 4), and L5 DRG after SNL (cross-hatched bar, n = 8). The data show the pronounced shift in favour of the short splice variant in L5 after SNL. Data are expressed as percentage of relative peak areas for ΔA+BΔC mRNA transcripts normalized to the sum of ΔA+B+C and ΔA+BΔC peak areas. Error bars represent SEM. ANOVA, F = 9.39, *P* = 0.000, and Gabriel post hoc analysis: ^∗∗^*P* < 0.01, ^∗∗∗^*P* < 0.001.

In order to detect and quantify any minor α_2_δ-1 splice variants in DRG, we used CE/LIF, and confirmed the presence of the major splice variant ΔA+B+C both in unaffected contralateral DRG (Fig. 3A), and in ligated DRG after SNL (Fig. 3B). The relative abundance of ΔA+B+C mRNA was quantified by CE/LIF after 27 cycles of RT-PCR (Fig. 3C). The ΔA+B+C-transcript level in ligated L5 showed a 3.6-fold increase, compared to the contralateral side. It was also significantly higher in L5 DRG following SNL, compared to L5 sham-operated animals, and compared to L4 SNL DRG and L4 DRG from sham-operated rats. There was no significant difference in α_2_δ-1 levels in L4 DRG between SNL and sham-operated animals (Fig. 3C).

Surprisingly, we also observed a novel splice variant in DRG neurons, ΔA+BΔC α_2_δ-1, which was particularly evident following the upregulation of α_2_δ-1 mRNA that occurs after L5/L6 SNL (Fig. 3B). ΔA+BΔC was identified in DRG both by DNA sequencing following agarose gel separation (unpublished results), and by the size of the product on CE/LIF after RT-PCR (Fig. 3A, B). The mRNA level of the ΔA+BΔC splice variant was quantified by CE/LIF after 29 cycles of RT-PCR (Fig. 3D). The expression level of ΔA+BΔC was found to be ∼10 times higher (1083%) on the ipsilateral side, compared to the respective contralateral side in L5 DRG. Moreover, ΔA+BΔC mRNA levels were significantly higher compared to L5 sham-operated animals, and L4 SNL and sham-operated rats. Although ΔA+BΔC mRNA levels were 2.4-fold higher in SNL L4 compared to L4 sham-operated animals, this was not statistically significant (*P* = 0.814).

Thus, the proportion of ΔA+B+C was significantly increased in L5, ipsilateral to SNL compared to sham-operated L5, but this was not the case for L4, which was not ligated. The ΔA+BΔC splice variant represented 5.1 ± 0.5% of the total α_2_δ-1 mRNA in sham-operated L5, and 8.4 ± 0.4% in SNL L5 DRG (Fig. 3E).

### 3.5 The relative abundance of ΔA+B+C and ΔA+BΔC in different DRG classes

DRG neurons are heterogeneous in morphology and the size of their somata relates to different functional subtypes. DRG with small and medium-sized somata form mainly nonmyelinated C fibres and myelinated Aδ fibres, which conduct pain sensation, whereas nonnociceptive Aβ fibres have larger somata [35]. α_2_δ-1 is expressed in every sub-type of DRG neuron [5], but to date, the distribution of α_2_δ-1 splice variants in DRG subpopulations is unknown. For the most prevalent DRG calcium channel α1 subunit, Ca_V_2.2, it has been found that there is a differential distribution of a particular splice variant in small DRG neurons [1], [6], and this splice variant is downregulated following SNL in rats [1]. Therefore, we wished to determine whether there was differential distribution in small and large DRG neurons of the α_2_δ-1 ΔA+BΔC splice variant, which showed reduced gabapentin binding affinity and a more pronounced upregulation following SNL, compared to the predominant α_2_δ-1 splice variant ΔA+B+C.

**Fig. 4:**
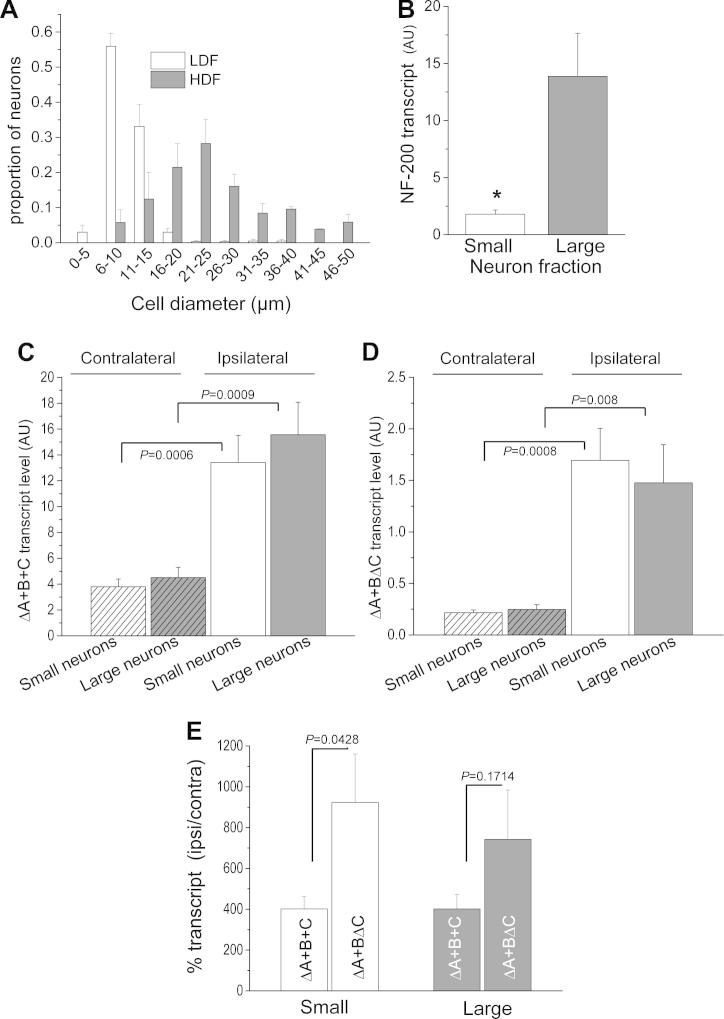
Analysis of the upregulation of ΔA+B+C and ΔA+BΔC in small and large dorsal root ganglion (DRG) neurons following spinal nerve ligation (SNL). (A) DRG neurons were separated into 2 populations (high-density fraction [HDF] and low-density fraction [LDF]) on the basis of size, by centrifugation through a Ficoll density gradient. The proportion of neurons as function of diameter in the fraction enriched in smaller neurons (LDF; white bars) and larger neurons (HDF; grey bars) is shown. Data are from 4 separate cultures obtained from the extraction of L5 and L6 DRG neurons from 2 naïve adult rats. When the same separation of DRG neurons was performed on SNL rats, all the material was used to extract the RNA, for use in capillary electrophoresis/laser-induced fluorescence (CE/LIF) experiments. A total of 418 cells from the LDF and 260 cells from the HDF were analysed. Error bars represent SEM. (B) Neurofilament-200 (NF-200) mRNA levels measured by CE/LIF are significantly higher in the HDF compared to the LDF. Data are expressed as NF-200 relative peak areas in the LDF (white bar, n = 4) and HDF (grey bar, n = 4). Error bars represent SEM. Statistical analysis was performed using an unpaired Student’s *t*-test, ^∗^*P* = 0.0187. (C, D) Transcript levels of α_2_δ-1 ΔA+B+C (C, n = 7) and ΔA+BΔC (D, n = 6) in small and large DRG neuron fractions prepared from L5/L6 DRG, ipsilateral and contralateral to SNL. Data are expressed as ΔA+B+C or ΔA+BΔC relative peak areas. Transcript levels in small (white bars) or large (grey bars) DRG neurons prepared from L5/L6 DRG, both contralateral (hatched bars) and ipsilateral (solid bars) to SNL. ΔA+B+C and ΔA+BΔC transcript levels were significantly higher on the ipsilateral side compared to the contralateral side in both the small neurons (4.0 ± 0.6-fold for ΔA+B+C and 9.2 ± 2.4-fold for ΔA+BΔC), and in the large neurons (4.0 ± 0.7-fold for ΔA+B+C and 7.4 ± 2.4-fold for ΔA+BΔC). Error bars represent SEM. Statistical analyses were performed using 2 sample unpaired Student’s *t*-test, for the data indicated. (E) Comparison of the upregulation in ipsilateral compared to contralateral L5 and L6 DRG of the 2 DRG α_2_δ-1 transcripts, 7 days after SNL. In the small neuron fraction (white bars), the extent of upregulation of ΔA+BΔC (right, n = 6) is significantly greater than that of ΔA+B+C (left, n = 7). In contrast, there is no significant difference between the upregulation of the 2 transcripts in the large neuron fraction (grey bars). Data are expressed as relative peak areas for the ipsilateral side normalized to the respective contralateral side. Error bars represent SEM. Statistical analyses were performed using unpaired Student’s *t*-test.

To obtain populations enriched with small or large neurons, DRG neurons extracted from L5 and L6 of 2 SNL rats were separated according to their size, as described in Materials and Methods. The LDF was enriched in smaller neurons, while the HDF was enriched in larger neurons (Fig. 4A). The mean diameter for neurons in the LDF was 10.0 ± 0.2 μm (n = 418), with a pronounced peak at 5–10 μm. In the HDF, the smallest neurons (<15 μm in diameter) were almost absent, and the neuron diameter was up to 50 μm, with a peak at 21–25 μm. The mean diameter was 24.5 ± 0.7 μm (n = 260). As expected, NF-200 mRNA, a marker for large myelinated DRG neurons [42], measured by CE/LIF, was significantly higher in the HDF (13.9 ± 3.8 arbitrary units (AU)) compared to the LDF (1.8 ± 0.4 AU) (Fig. 4B), confirming that the HDF contains mainly larger neurons expressing NF-200.

We then used RT-PCR and CE/LIF to quantify the relative abundance of α_2_δ-1 ΔA+B+C and ΔA+BΔC in the small and large DRG neuron fractions after SNL. In agreement with the previous experiments, both ΔA+B+C (Fig. 4C) and ΔA+BΔC (Fig. 4D) transcript levels were significantly increased on the ipsilateral side compared to the contralateral side after SNL. We further analysed whether the differential upregulation of ΔA+BΔC compared to ΔA+B+C mRNA found in whole DRG (Fig. 3E) was present in both small and large neuron fractions. This was calculated as the percentage increase on the ipsilateral compared to the contralateral side, of the 2 transcripts in the small neuron fraction (white bars) compared to the large neuron fraction (grey bars). This analysis was performed to provide a direct comparison with the values found in whole DRG (Fig. 3C, D). We found that the increase of ΔA+BΔC mRNA following SNL was significantly greater compared to the corresponding ΔA+B+C increase, only in the small neuron fraction, and not in the large neuron fraction (Fig. 4E).

Thus, although upregulation following SNL of the 2 DRG transcripts ΔA+B+C and ΔA+BΔC occurs in both small and large DRG neurons (Fig. 4C, D), the increased upregulation of ΔA+BΔC compared to ΔA+B+C following SNL occurs preferentially in the small DRG neuron fraction containing nonmyelinated nociceptors (Fig. 4E).

### 3.6 Electrophysiological properties of the 2 splice variants present in DRG

**Fig. 5:**
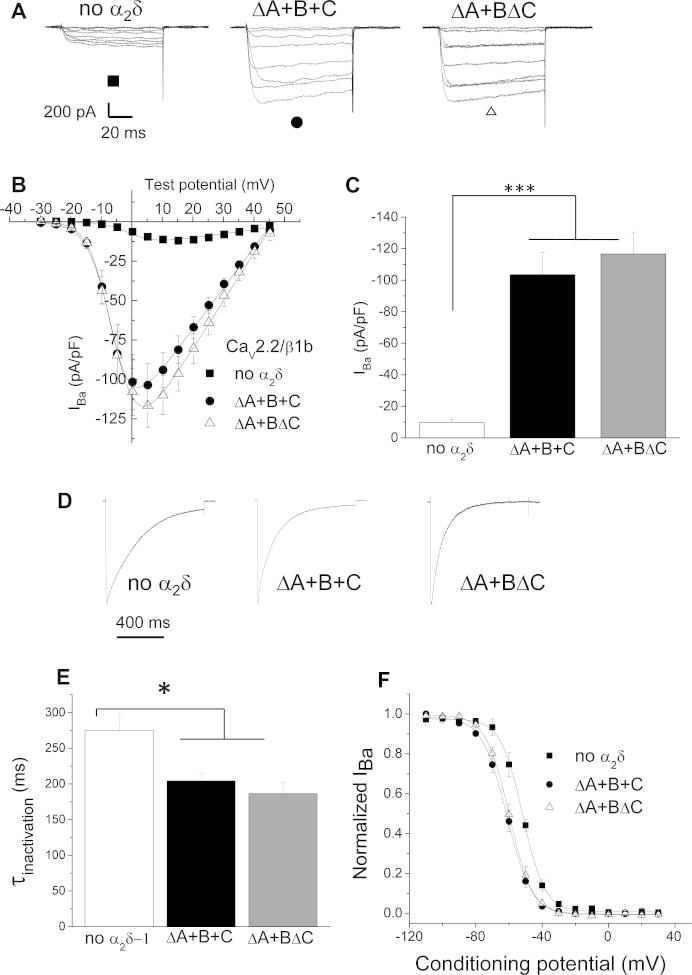
Comparison of the effect of the 2 main dorsal root ganglion α_2_δ-1 splice variants on Ca_V_2.2 calcium channel current properties. (A) Representative current traces elicited between −30 and +40 mV in 10-mV voltage steps from a holding potential of −90 mV for Ca_V_2.2/β1b, co-expressed in tsA-201 cells either without α_2_δ (left, ■), or with α_2_δ-1 ΔA+B+C (middle, ●) or α_2_δ-1 ΔA+BΔC (right, Δ). 1 mM Ba^2+^ was used as the charge carrier. (B) Current-voltage (I-V) relationships for the 3 experimental conditions: Ca_V_2.2/β1b/ α_2_δ-1 ΔA+B+C (●, n = 16), Ca_V_2.2/β1b/ α_2_δ-1 ΔA+BΔC (Δ, n = 20), or Ca_V_2.2/β1b (■, n = 12). Current amplitude was normalized to whole-cell capacitance and plotted against membrane potential. Data are fitted using a modified Bolzmann function, as previously described [12]. (C) Peak current density was −103.56 ± 13.9 pA/pF in presence of ΔA+B+C (black bar) and −116.63 ± 13.9 pA/pF in presence of ΔA+BΔC (grey bar). These values were both significantly higher than the current density measured in the absence of α_2_δ-1, which was 9.6 ± 2 pA/pF (white bar). Error bars represent SEM. Statistical analysis was performed using 1-way analysis of variance (ANOVA) and Bonferroni post hoc analysis, ^∗∗∗^ denotes *P* < 0.001). (D) Representative current traces in response to a long depolarizing voltage step (0.9 second) to 0 mV for Ca_V_ 2.2/β1b, co-expressed in tsA-201 cells either without α_2_δ (left), or with α_2_δ-1 ΔA+B+C (middle) or α_2_δ-1 ΔA+BΔC (right). 1 mM Ba^2+^ was used as a charge carrier. Holding potential was −90 mV. Traces are normalized to their peak. (E) α_2_δ-1 ΔA+B+C and ΔA+BΔC significantly accelerated the inactivation of currents compared to no α_2_δ. The decay phase of individual current traces at 0 mV was fitted with a single exponential function, and the mean time constant (τ) of inactivation was 204.3 ± 10.3 ms for ΔA+B+C (black bar, n = 10), 186.4 ± 15.8 ms for ΔA+BΔC (grey bar, n = 14), and 248.0 ± 23.6 ms for Ca_V_2.2/β1b without α_2_δ (white bar, n = 10). Statistical analyses were performed using 1-way ANOVA, Bonferroni post hoc analysis, ^∗^*P* < 0.05. (F) Steady-state inactivation curves for I_Ba_ evoked by a test pulse to +20 mV after a 10-second conditioning prepulse of between −100 and 0 mV. Ca_V_2.2/β1b/α_2_δ-1 ΔA+B+C (●, n = 15), Ca_V_2.2/ β1b/α_2_δ-1 ΔA+BΔC (Δ, n = 13), Ca_V_2.2/ β1b (■, n = 10). Error bars represent SEM. Data were fitted with a single Boltzmann equation, and the mean voltages at which the channel is 50% inactivated were −61.3 ± 1.2 mV, −60.0 ± 1.9 mV, and −50.7 ± 1.9 mV, respectively.

It was important to determine whether α_2_δ-1 ΔA+BΔC was a functional splice variant, and we therefore compared the properties of Ca_V_2.2 calcium channel currents co-expressed with β1b and either α_2_δ-1 ΔA+BΔC or ΔA+B+C. Ca_V_2.2 was used because it is the main calcium channel in DRG neurons. We found that the properties of the currents formed from these combinations were very similar in terms of their ability to increase Ca_V_2.2 current density compared to the absence of α_2_δ (Fig. 5A–C), their ability to increase the inactivation rate of the currents (Fig. 5D, E), and their ability to hyperpolarize the steady-state inactivation of the currents (Fig. 5F). Since Ca_V_2.1 is also present in DRG neurons, we also examined calcium currents formed by this channel. Similar results were obtained, in terms of the effect of the α_2_δ-1 splice variants on current amplitude (Supplementary Fig. 2A–C), voltage-dependence of inactivation (Supplementary Fig. 2D), and kinetics of inactivation (Supplementary Fig. 2E).

## 4 Discussion

### 4.1 Genomic arrangement of α_2_δ-1 giving rise to splice variants

The *cacna2d1* gene encoding mouse α_2_δ-1 was found to contain 3 alternatively spliced regions within the α_2_ moiety (A, B, and C) [3]. It was originally reported that regions A and B were part of the same exon, with alternative 3′ splice acceptor site, which spliced region A in or out [3]. In this scenario, splice variants of α_2_δ-1 should not exist in which region A is expressed without B. However, our analysis of the mouse genomic sequence has found that A is encoded by exon 18a, and B is formed as a result of utilizing an alternative 3′ splice acceptor site for exon 19. In confirmation of this, we have found splice variants in rat heart in which A is expressed without B (Fig. 1C).

In skeletal muscle, α_2_δ-1 +A+BΔC was the only splice variant detected [3]. We did not identify any minor splice variants in skeletal muscle in our study. We also found that the +A+BΔC splice variant bound ^3^H-gabapentin with high affinity, in agreement with previous findings of high-affinity binding of ^3^H-gabapentin to native rat skeletal muscle α_2_δ-1 [27]. Our results confirm that the lack of effect of gabapentin on skeletal muscle function is not a result of its inability to bind to the skeletal muscle α_2_δ-1 isoform.

In mouse heart, α_2_δ-1 ΔAΔB+C was previously found to be the most prevalent splice variant, although other splice variants were also observed, specifically ΔA+B+C, ΔAΔBΔC, and ΔA+BΔC [3]. In this study we found that ΔAΔBΔC was the predominant isoform in rat heart tissue, but we also found 6 other splice variants (Fig. 1C). It is of interest to note that the ΔAΔBΔC splice variant has a significantly lower affinity for ^3^H-gabapentin, compared to the major brain splice variant (Fig. 2). This result correlates with an early finding that the specific binding of ^3^H-gabapentin to heart membranes is lower than that in brain membranes, although this may additionally represent a smaller number of binding sites [27]. In brain, the only splice variant found previously was ΔA+B+C [3], and its presence was confirmed in our study. We found that this was also the main splice variant in DRG neurons.

### 4.2 Differential upregulation of the minor α_2_δ-1 splice variant in DRG after SNL

Following SNL in rats, we found previously that the level of α_2_δ-1 mRNA was increased >500% in L5/L6 DRG neurons, ipsilateral compared to contralateral to the ligation, and this was accompanied by a similar increase in α_2_δ-1 protein [5]. We confirmed that result here, for the main splice variant ΔA+B+C, where a 3.6-fold increase was observed. However, by CE/LIF we were also able to observe the presence in DRG of a second, minor splice variant ΔA+BΔC, whose level was increased over 10-fold following SNL. Therefore, this splice variant, which has a lower binding affinity for ^3^H-gabapentin, was differentially upregulated in SNL. In the experiments described here, ΔA+BΔC increased from 5.1% of the total transcript in sham-operated L5 DRG to 8.4% in L5 ipsilateral to SNL.

### 4.3 Involvement of spliced regions of α_2_δ-1 in ^3^H-gabapentin binding

To date, several sites in α_2_δ-1 have been established to be important for its ability to bind gabapentin [9], [50]. The triple arginine motif, situated just upstream of the VWA domain in α_2_δ-1, is essential for high-affinity ^3^H-gabapentin binding, and also for the ability of gabapentin to alleviate hyperalgesia [24], and to inhibit calcium currents when applied chronically [30]. One of the other regions identified by Wang et al. [50] is located just downstream of region B, and another is just upstream of region C.

In the present study, we found that both the ΔA+BΔC and ΔAΔBΔC splice variants possessed significantly lower affinity for ^3^H-gabapentin, compared to the other splice variants tested. However, there was no significant difference in the gabapentin binding affinity to α_2_δ-1 +A+B+C, containing region A, compared to its absence in ΔA+B+C. Similarly, for the presence or absence of region B, the ^3^H-gabapentin binding affinity was similar for α_2_δ-1 ΔA+B+C and ΔAΔB+C. These data indicate that A and B are not directly involved in the binding of gabapentin. This suggests that it is primarily the absence of region C in α_2_δ-1 ΔA+BΔC and ΔAΔBΔC that is critical for the reduced ^3^H-gabapentin binding affinity of these 2 splice variants. The sequence of region C in rat α_2_δ-1 is SKKGKMK, which is positively charged, as is the triple arginine motif that is essential for ^3^H-gabapentin binding.

It is notable that the affinity of gabapentin for α_2_δ-1 is much greater (ie, a lower K_D_) than the clinically relevant concentrations. One potential factor contributing to this discrepancy is that an endogenous ligand may occupy the gabapentin-binding site on α_2_δ-1 *in vivo*, and compete with gabapentin. This also accounts for the finding that purification of the α_2_δ proteins results in a marked increase in apparent affinity for gabapentin, as the endogenous ligand is removed during purification [8], [18].

### 4.4 Efficacy of gabapentin following SNL

It has been demonstrated that the effect of gabapentin is state dependent, in that in most circumstances it is able to inhibit neuropathic pain responses in experimental animals, while having no effect on physiological nociception [14], [24], [25], [41]. Alterations in gene expression occur following neuropathic nerve damage, which lead to changes in primary afferent inputs into the spinal cord, including the upregulation of α_2_δ-1 in these terminals [5]. It has been demonstrated that gabapentin blocks calcium currents acutely in DRG from mice overexpressing α_2_δ-1, but not in control animals [36], and this model may mimic the neuropathic condition. Our results indicate that the upregulation of specific splice variants of α_2_δ-1 in SNL does not contribute to determining the state dependence of the efficacy of gabapentin, since no splice variants with an increased affinity for gabapentin were identified in DRG neurons following SNL. Thus, the state dependence of gabapentin is likely to depend on the overall elevation of all α_2_δ-1 splice variants.

### 4.5 Biophysical properties of α_2_δ-1 splice variants found in DRG

It is thought that increased presynaptic calcium currents, resulting from the upregulation of α_2_δ-1, contribute to the hyperexcitability of DRG that underlies hyperalgesia and allodynia [36]. If the alternative splicing of α_2_δ-1 were to modify the electrophysiological properties of α_2_δ-1-containing channels, the consequences of altered function would be augmented following differential upregulation of the α_2_δ-1 ΔA+BΔC splice variant, as a consequence of nerve damage. However, we have demonstrated here that the ability of α_2_δ-1 ΔA+BΔC to elicit calcium currents is not affected by deletion of region C. α_2_δ-1 ΔA+BΔC enhances calcium currents resulting from the co-expression with either Ca_V_2.2 or Ca_V_2.1 to the same extent as α_2_δ-1 ΔA+B+C. This result indicates that the differential upregulation of α_2_δ-1 ΔA+BΔC in DRG neurons does not contribute to triggering hyperexcitability to a greater extent than the main DRG splice variant.

### 4.6 Changes in calcium channel splicing following sensory nerve damage

It is of interest to compare our results with those for the exon 37a variant of Ca_V_2.2, which is selectively expressed in DRG neurons, and conducts larger calcium currents than the exon 37b variant [1], [6]. Despite being present overall at ∼7% of the main splice variant 37b of Ca_V_2.2, it was found selectively in small DRG neurons also expressing VR1 [1], [6]. Interestingly, the 37a splice variant was downregulated to ∼2% in DRG following SNL [1], whereas there was no change in the main Ca_V_2.2 splice variant containing exon 37b.

In contrast, in our study there was a greater increase of α_2_δ-1 ΔA+BΔC compared to the main splice variant α_2_δ-1 ΔA+B+C following SNL, and this differential increase occurred particularly in the nonmyelinated small DRG neuron fraction. This, together with our finding that ΔA+BΔC had a significantly lower affinity for gabapentin, and an equivalent ability to enhance neuronal calcium currents, could be highly relevant to the response to gabapentin in patients with chronic neuropathic pain. The rat α_2_δ-1 ΔA+BΔC mRNA sequence used in this study has 95% amino acid homology with the human sequence, and C region is identical in both species. Therefore, it is reasonable to speculate that human α_2_δ-1 ΔA+BΔC (accession number P54289 isoform 5) is expressed in DRG neurons and undergoes similar upregulation in conditions leading to the development of chronic neuropathic pain. It is, further, tempting to speculate that differences in the extent of upregulation of α_2_δ-1 ΔA+BΔC in humans could potentially contribute to the variable efficacy of gabapentinoid drugs [37], [38].

### 4.7 Conclusion

We have identified a novel α_2_δ-1 splice variant ΔA+BΔC, which is expressed in DRG neurons and is differentially upregulated compared to the main DRG splice variant α_2_δ-1 ΔA+B+C, particularly in small nonmyelinated DRG neurons following SNL. This splice variant supports Ca_V_2 calcium currents with unaltered properties, but shows a significantly reduced affinity for gabapentin. It could therefore play a role in determining the efficacy of gabapentin in different forms of neuropathic pain. Furthermore, targeting this splice variant for drug discovery could increase the therapeutic efficacy of gabapentinoid drugs in the future.

## Conflict of interest statement

The authors have no conflicts of interest to report.

## Appendix A Supplementary data

Supplementary data 1 This document contains Supplementary Figs. 1 and 2.

Supplementary data 2 This document contains Supplementary Figs. 1 and 2 captions.
