# Supplementary figures and images for: Differential upregulation in DRG neurons of an α2δ-1 splice variant with a lower affinity for gabapentin after peripheral sensory nerve injury
Source: Pain. 2014 Mar;155(3):522–33. doi: 10.1016/j.pain.2013.12.001 (PMC3988960; doi:10.1016/j.pain.2013.12.001)

A

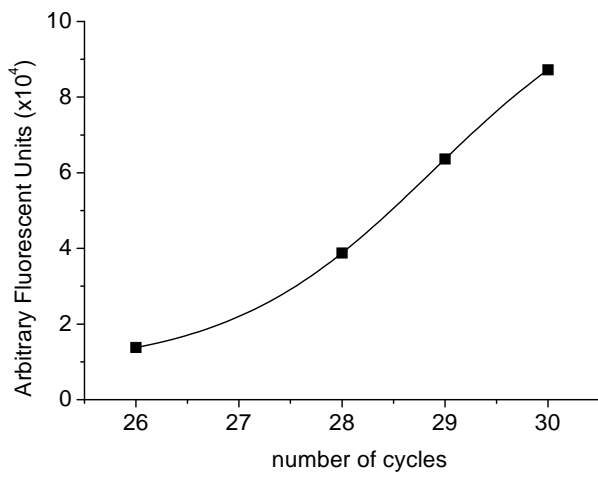

B

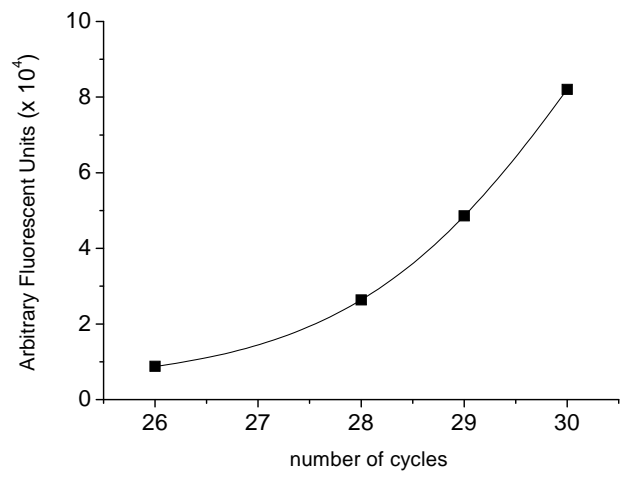

C

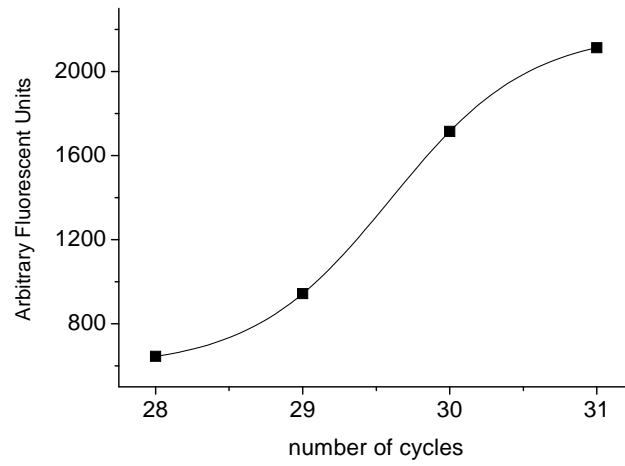

D

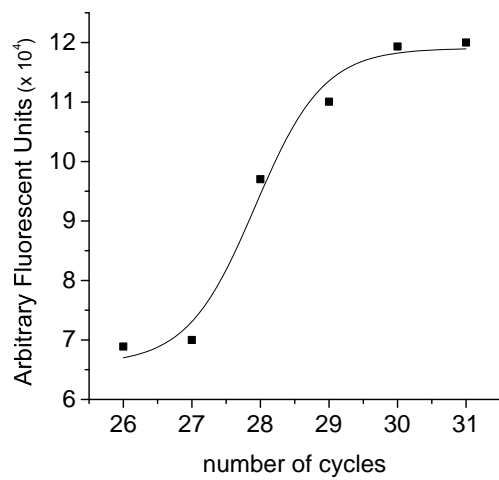

E

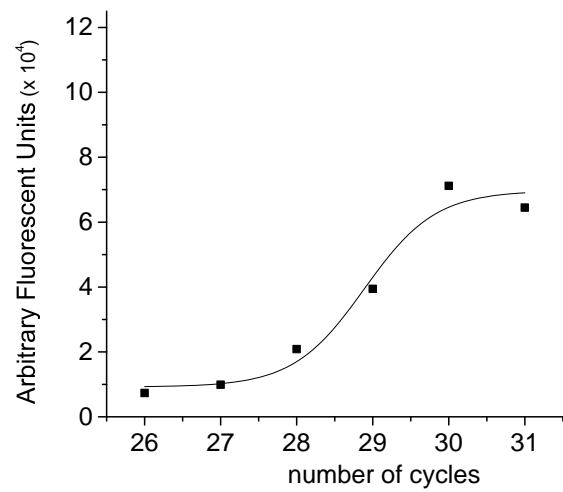

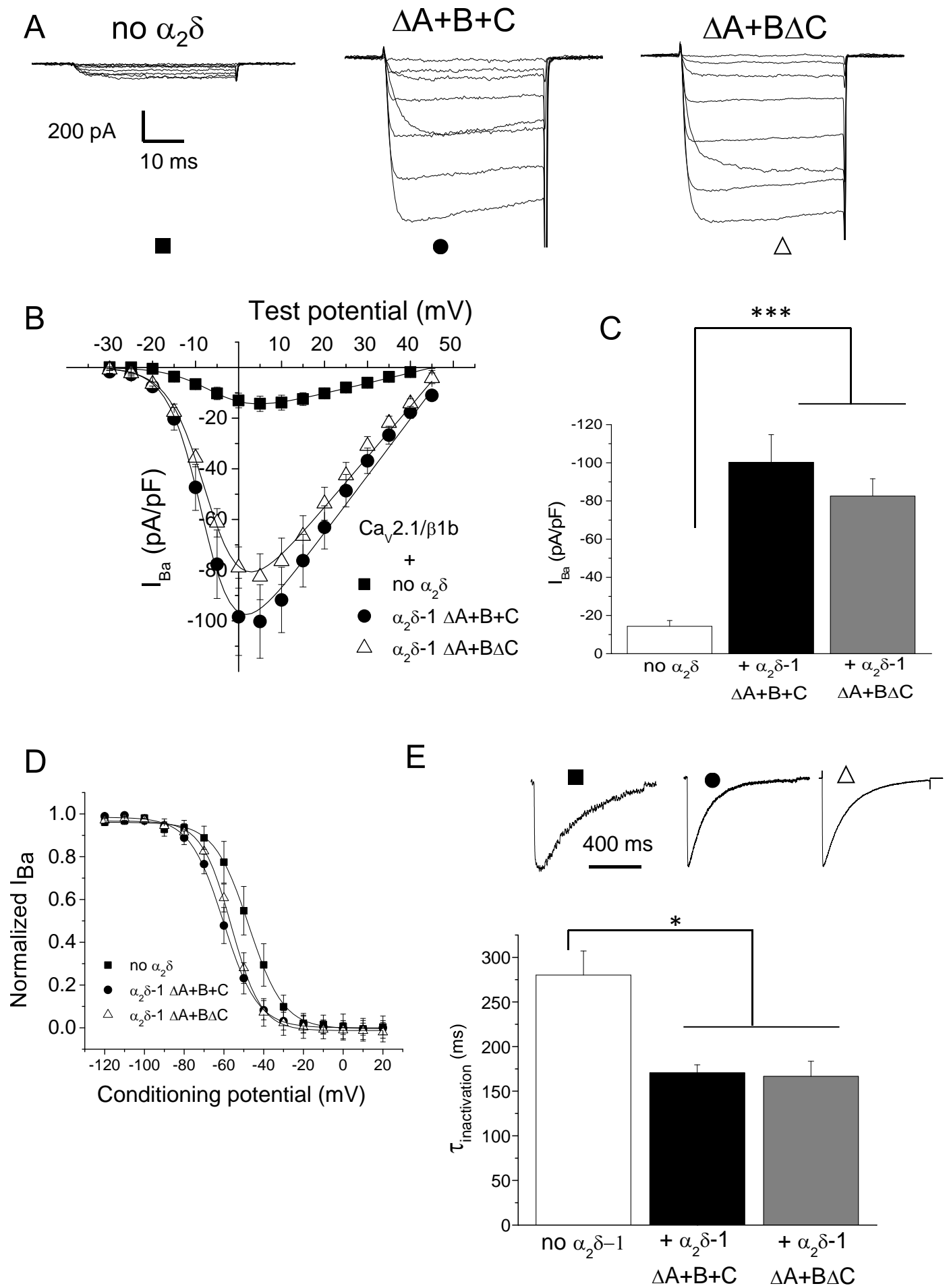

Supplement: Supplementary data 1 — This document contains Supplementary Figs. 1 and 2. [file mmc1.pdf]
