# Supplementary material for: Differential upregulation in DRG neurons of an α2δ-1 splice variant with a lower affinity for gabapentin after peripheral sensory nerve injury
Source: Pain. 2014 Mar;155(3):522–33. doi: 10.1016/j.pain.2013.12.001 (PMC3988960; doi:10.1016/j.pain.2013.12.001)
Supplement: Supplementary data 2 — This document contains Supplementary Figs. 1 and 2 captions. [file mmc2.docx]

**Supplementary Figure legends**

**Supplementary Fig. 1: Amplification reaction kinetics of multiplex PCR.**

The figure depicts plots of integrated peak areas vs cycle number for (A) TBP (144 bp) and α_2_δ-1 splice variants (B) ΔA+B+C (381 bp) and (C) ΔA+BΔC (360 bp). RT-PCR was performed on 100ng of total RNA equivalent, derived from L5 DRGs of SNL rats. Target sequences and housekeeping gene (TBP) were co-amplified in a single reaction. The same experiment was performed to optimize the linearity of (D) NF-200 (99 bp) and (E) TBP (144 bp) in LDF and HDF of DRG neurons. RT-PCR was performed on 4ng of total RNA equivalent, derived from L5 and L6 DRGs of naïve rats. Amplicon quantity was determined by CE/LIF at the completion of an increasing number of thermal cycles. This result shows that data were collected and analyzed within the exponential range of amplification. Data were fit using a sigmoid curve.

**Supplementary Fig. 2: Comparison of the effect of ΔA+BΔC and ΔA+B+C of α_2_δ-1 on Ca_V_ 2.1 currents in tsA-201 cells**

A-F. Experiments shown in Figs. 6 and 7 were repeated following co-transfection of tsA-201 cells with α_2_δ-1 ΔA+B+C or ΔA+BΔC, β1b and Ca_V_2.1, instead of Ca_V_2.2. 1mM Ba^2+^ was used as charge carrier. A: Representative current traces elicited between -30 and +40 mV in 10 mV voltage steps from a holding potential of – 90 mV. B: I-V relationships for Ca_V_2.1/β1b/α_2_δ-1 ΔA+B+C (●, n=17), Ca_V_2.2/β1b/α_2_δ-1 ΔA+BΔC (∆, n=21) and Ca_V_2.1/β1b (■, n=12). Current amplitude was normalized to whole-cell capacitance and plotted against membrane potential. The curves are fit with a modified Bolzmann function. C: The measured current densities were -100.2 ± 14.5 pA/pF for ΔA+B+C (black bar), -82.6 ± 9.0 pA/pF for ΔA+BΔC (gray bar) and -14.3 ± 3.0 pA/pF for Ca_V_2.1/β1b without α_2_δ (white bar,). D: Steady-state inactivation curve for a test pulse to +20 mV following 10 s conditioning pre-pulses between -100 and 0 mV. Ca_V_2.1/β1b/α_2_δ-1 ΔA+B+C (●, n=17), Ca_V_ 2.1/β1b/α_2_δ-1 ΔA+BΔC (∆, n=10), Ca_V_2.1/β1b without α_2_δ-1 (■, n=6). Data were fitted with a single Boltzmann equation and the mean voltages at which the channel is 50% inactivated were -59. 7 ± 1.3 mV, -58.0 ± 1.7 mV and -47.8 ± 3.9 mV, respectively. E: Representative current traces in response to a long depolarizing voltage step (0.9 s) to 0 mV for Ca_V_2.1/β1b (■, left), Ca_V_2.1/β1b/α_2_δ-1 ΔA+BΔC (●, middle) and Ca_V_2.1/β1b/α_2_δ-1 ΔA+B+C (∆, right). Holding potential was -90 mV. Traces are normalized to their peak. The decay phase of individual current traces at 0 mV was fitted with a single exponential function and the mean time constants of inactivation (τ_inactivation_) were 280.1 ± 27.1 ms for Ca_V_ 2.2/β1b (white bar, n=10), 170.6 ± 27.1 ms for ΔA+B+C (black bar, n=18), 166.7 ± 16.9 for ΔA+BΔC (gray bar, n=11). Error bars represent SEM. Statistical analyses were performed using one-way ANOVA and Bonferroni post-hoc analysis, *denotes *P*<0.05.
